# Depression and anxiety symptoms among Vietnamese migrants in Japan during the COVID-19 pandemic

**DOI:** 10.1186/s41182-023-00542-8

**Published:** 2023-10-31

**Authors:** Tadashi Yamashita, Pham Nguyen Quy, Emi Nogami, Erina Seto-Suh, Chika Yamada, Saori Iwamoto, Kyoko Shimazawa, Kenji Kato

**Affiliations:** 1 Faculty of Nursing, Kobe City College of Nursing, 3-4 Gakuennishi-Machi, Nishi-ku, Kobe, Hyogo 651-2103 Japan; 2 Department of Medical Oncology, Kyoto Miniren Central Hospital, Kyoto, Japan; 3 Department of Social Welfare, School of Psychology and Social Welfare, Mukogawa Women’s University, Nishinomiya, Japan; 4 Human Rights Research Institute, Kindai University, Higashi Osaka, Japan; 5 Department of Environmental Coexistence, Center for Southeast Asian Studies, Kyoto University, Kyoto, Japan; 6 Faculty of Global Nursing, Otemae University, Osaka, Japan; 7 Faculty of Nursing, Kobe Women’s University, Kobe, Japan

**Keywords:** Mental health, Depression, Anxiety, Migrants, Vietnamese, COVID-19

## Abstract

This study aimed to examine the mental health status and related factors among Vietnamese migrants in Japan during the COVID-19 pandemic. We conducted an online cross-sectional survey between September 21 and October 21, 2021. Along with Patient Health Questionnaire-9 (PHQ-9) and Generalized Anxiety Disorder 7-item (GAD-7) scores, we collected data on demographics, changes in socioeconomic status due to the pandemic, language proficiency, social support, and health conditions. Multivariate logistic regression was performed to identify factors related to symptoms of depression and anxiety. Among 621 participants who completed the questionnaire, moderate-to-severe symptoms of depression (PHQ-9 score ≥ 10 points) and mild-to-severe symptoms of anxiety (GAD-7 score ≥ 5 points) were observed in 203 (32.7%) and 285 (45.9%) individuals, respectively. Factors related to depressive symptoms were age (95% confidence interval [CI]=0.89-0.99), pre-existing health conditions (95% [CI]=1.61–3.76), and a low subjective socioeconomic status (95% [CI]=1.64–3.71). Factors related to anxiety symptoms were being single (95% [CI]=1.01–2.93), having pre-existing health conditions (95% [CI]=1.63–3.88), subjective socioeconomic status (95% [CI]=1.87–3.97), and absence of a partner to discuss one’s health with (95% [CI]=1.11–2.47). Vietnamese migrants in Japan experienced a decrease in income, worsening working conditions, and poor mental health status during the COVID-19 pandemic. Further investigations are necessary to find an effective way to increase their social support and mitigate socioeconomic adversities.

## Background

The number of foreign residents in Japan has continued to increase annually, totalling approximately 2,960,000 as of June 2022. One notable group within this population is Vietnamese migrants, whose numbers have grown from 10,000 in 1990 to 470,000 in 2022 [1]. A more recent rise in migration attributed to an increase in the number of Vietnamese technical interns, skilled laborers, and students [2].

World Health Organization (WHO) reported that migrants are at a risk for impaired mental health due to traumatic and stressful experiences and being possible be at an increased risk for depression and anxiety. The COVID-19 pandemic is thought to have exacerbated inequalities in immigration as a cause of mental health problems [3]. In 2020, a large online survey was conducted in 37 languages including 20,742 migrants from around the world. The results showed that depression was present in 50.9% and anxiety in 49.2%. Mental deterioration was particularly prevalent in unstable housing and residential situations, among older respondents, and among women [4]. The pandemic also affected the lives of migrants in Japan, who already hold a marginalized and precarious position, due to reduced working hours and increased layoffs. A survey on mental health of migrants reported that most students from China, living in Japan, have worries concerning COVID-19 prevention [5], and approximately 70% of Chinese migrants in Japan have expressed concerns regarding COVID-19 infection [6]. However, the mental health status of Vietnamese migrants in Japan during the COVID-19 pandemic remains unclear. The mental health of Vietnamese migrants living in Japan being possible associated with changes in lifestyle and social functioning because of the COVID-19 pandemic. The mental health of the second-largest foreign community in Japan—remains an important public health concern. Therefore, to identify factors being possible influence mental health status, the present study aimed to investigate symptoms of depression and anxiety among Vietnamese migrants in Japan during the COVID-19 pandemic.

## Methods

### Design and sampling

This study was conducted in accordance with the tenets of the Declaration of Helsinki. This cross-sectional survey was conducted among Vietnamese migrants in Japan from September 21 to October 21, 2021. During the survey, Japan was in the latter half of the fifth COVID-19 wave. To prevent COVID-19 spread through contact, we conducted an online survey using SurveyMonkey (Momentive Inc., San Mateo, CA, USA).

We conducted the survey using social networks of Facebook as the distribution platform. Young Vietnamese are reported to use social media such as Facebook daily [7, 8]. A one-page participants information statement, which also served as a recruitment poster, was posted in Facebook groups. This statement briefly explained the study background, purpose, and procedure; voluntary nature of participation; anonymity of the questionnaire; strict privacy protection practices; and how to complete the questionnaire. To further recruit Vietnamese in Japan, who do not use social networking services, we invited them to participate in the survey at churches that regularly visited by the Vietnamese migrants in Japan. The inclusion criteria for participation were as follows: Vietnamese or Japanese citizenship in those of Vietnamese descent, current residence in Japan, and age ≥ 18 years. To avoid duplicate responses from participants, SurveyMonkey was used to allow only one response from the same terminal.

### Data collection tools

The survey involved a self-administered questionnaire, which was used to collect data regarding demographic variables, such as sex, age, duration of residence in Japan, area of residence, marital status, education level, birth country, visa status, Japanese language level, and pre-existing health conditions. Based on the opinions of Japanese infectious disease experts, we also classified residence into three categories based on the number of COVID-19 infections as of December 2021 (> 2,000 per 100,000: Tokyo, Osaka, Okinawa; 1,999–1,000 per 100,000: Hokkaido, Saitama, Chiba, Kanagawa, Aichi, Kyoto, Nara, Hyogo, Fukuoka; < 999 per 100,000: Others).

The economic and employment status of the participants was assessed using multiple indicators, including national health insurance coverage, public assistance, subjective socioeconomic status, and monthly income compared to pre-COVID-19 levels. Under Japanese law, international students are allowed to work part-time or up to 28 h per week outside their visa status. This income may account for a large portion of their living expenses. Therefore, in this survey, we also ascertained the income and working conditions of international students. Social support status was assessed based on whether patients had partners with whom they can talk regarding their health. The levels of depression and anxiety were assessed using the Patient Health Questionnaire-9 (PHQ-9) and Generalized Anxiety Disorder seven-item (GAD-7), respectively, which have been validated for assessing mental health status in Vietnam [9–11]. In our study, the Cronbach's alpha values for the reliability of the PHQ-9 and GAD-7 were 0.844 and 0.933, respectively.

### Statistical analysis

We performed descriptive analyses using means and standard deviations (SD) for continuous variables as well as counts and percentages for binary or categorical variables. Each PHQ-9 item was rated on a four-point Likert scale ranging from 0 (not at all) to 3 (almost every day), with the total score ranging from 0 to 27 points. A PHQ-9 score ≥ 10 points was considered to indicate clinically relevant depressive symptoms, based on a previous survey of COVID-19 in Vietnam [10]. Each question item ranges from 0 (not at all) to 3 (almost every day) on a 4-point Likert scale, and the total score ranged from 0 to 21 points. GAD-7 scores ≥ 5 points were considered to indicate clinically relevant symptoms of anxiety, as previously reported [9, 11].

We conducted multivariable logistic regression analysis (forced-entry method) to identify factors related to symptoms of depression and anxiety, which were used as the dependent variables. The potential predictors included sociodemographic variables, such as age, sex, marital status, education level, pre-existing health conditions; migrants’ specific variables, such as the duration of residence in Japan, visa status, Japanese language proficiency; economic variables; variables related to the COVID-19 pandemic, such as the rate of COVID-19 infection in the area; and variables related to social connectedness, such as the availability of a conversation partner. In this analysis, economic status, not employment status, was used as a predictor because employment status affects economic status. Inclusion of variables was based on previous studies of mental health among migrants [12, 13], and the chosen variables were entered simultaneously for multivariable regression. We created a correlation matrix before entering the independent variables; further, we confirmed that there were no strong correlations among the independent variables with *r* > 0.80. To assess the goodness of fit of the logistic regression model for the PHQ-9 and GAD-7, we conducted a χ2 test with an omnibus test of model coefficients. The results demonstrated a significance level of p < 0.001, indicating that the model was considered significant. To further validate the goodness of fit of the multivariable logistic regression model, we performed the Hosmer and Lemeshow test. The p-values for the PHQ-9 and GAD-7 scores were 0.671 and 0.194, respectively, with p ≥ 0.05. These findings indicate that the model exhibited an acceptable level of goodness of fit. Moreover, an unadjusted bivariate model was employed to assess the possibility of over-control or over-adjustment in the multivariable logistic regression model. Statistical analyses were performed using the SPSS software (version 19.0; IBM Corp., Armonk, NY, USA). Statistical significance was set at p < 0.05 (two-sided tests).

## Results

A total of 1,046 people participated in the online survey, with 652 participants providing complete responses. After deleting responses with missing values or outliers, we included 621 responses. All participants were Vietnamese. The mean age (± SD) was 26.0 ± 4.8 years; further, the mean duration of residence in Japan (± SD) was 3.4 ± 3.1 years. Approximately 30% of the respondents lived in areas with high infection rates. Most respondents (79.2%) were single (including divorced or bereaved), and almost all were born in Vietnam (99.0%). The most common visa status was "technical intern training (Ginojisshu)" (29.5%), followed by "international student" (29.3%) and "status of residence based on employment" (26.9%). The most common educational level was high school (38.3%), followed by college or university (34.5%) and technical school (21.3%). Further, 21.3% of respondents had a pre-existing health conditions, and the most common was gastroenterology (7.9%), followed by otorhinolaryngology (5.2%) and orthopedics (5.2%) (Table 1).

**Table 1:** Participant characteristics (n = 621)

	n	%
Sex		
Male	347	55.9
Female	274	44.1
Age (year)		
(Mean ± SD)	26.0 ± 4.8	
Duration of residence in Japan (year)		
(Mean ± SD)	3.4 ± 3.1	
COVID-19 Infection rate in the area of residence		
Low	181	29.1
Middle	251	40.4
High	189	30.4
Marital status		
Married	129	20.8
Single, divorced, or bereaved	492	79.2
Visa status		
Residence based on the nature of activities	26	4.2
Residence based on employment except Technical Intern Training and Skilled Labor	167	26.9
International student	182	29.3
Technical Intern Training (Ginojisshu)	183	29.5
Skilled Labor (Tokuteiginou)	38	6.1
No response	25	4.0
Japanese language proficiency		
Ability to speak fluently and confidently	71	11.4
Ability to speak at a level that does not affect work or study	273	44.0
Adequate ability to speak without impacting daily life	217	34.9
Barely able to speak Japanese	60	9.7
Education level		
Junior high school and under	5	0.8
High school	238	38.3
Technical school	132	21.3
College (2 years) or University	214	34.5
Graduate school	32	5.2
Pre-existing health conditions		
Yes	132	21.3
No	489	78.7
Pre-existing health conditions (Multiple answers)		
Gastroenterology	49	7.9
Otorhinolaryngology	32	5.2
Orthopedics	32	5.2
Dentistry	31	5
Psychiatry	25	4
Cardiology	23	3.6
Urology	17	2.7
Hematology	12	1.9
Gynecology	8	1.3
Respiratory	4	0.6
Endocrinology	3	0.5
Neurology	3	0.5
Oncology	2	0.3

Regarding economic status, 5.5% and 6.9% of respondents lacked national health insurance and used public assistance, respectively. 34.6% of respondents reported a decrease in monthly income when compared with the period before the COVID-19 pandemic, with 39.1% reporting a slight decrease. 14.2% and 46.2% of participants reported very poor and poor subjective socioeconomic status, respectively. Additionally, 18.7% of respondents reported dismissal from work/unemployment during the COVID-19 pandemic, while 64.1% reported a reduction in the number of working days (Table 2).

**Table 2:** Economic and employment status (n = 621)

	n	%
National health insurance		
Yes	587	94.5
No	34	5.5
Public assistance		
Used	43	6.9
Not used	578	93.1
Monthly income when compared with that before COVID-19		
Decrease of ≥ 40%	215	34.6
Decrease of 10–40%	243	39.1
Decrease < 10%	146	23.5
Increase > 10–40%	15	2.4
Increase > 40%	2	0.3
Subjective socioeconomic status		
Really good	5	0.8
Slightly better	47	7.6
General	194	31.2
Slightly difficult	287	46.2
Very difficult	88	14.2
Employment status (multiple answers)		
No change/no impact	93	15.0
Dismissal/Unemployment	116	18.7
Change in occupation type	76	12.2
Change in work content	101	16.3
Reduction in working days	398	64.1
Increase in working days	17	2.7
Other	44	7.1

*International students were included in the statistical analysis for “Economic and employment status” since they are allowed to have part-time jobs in Japan

Further, 30.3% of participants reported having a partner to discuss their health with, with family (21.9%) being the most common, followed by Vietnamese friends (9.4%), colleagues (8.7%), and medical professionals (including through online consultation) (4.3%) (Table 3).

**Table 3:** Social support status (n = 621)

	n	%
Partner to discuss health status with		
Yes	188	30.3
No	433	69.7
Who is the partner with whom you can discuss your health? (Multiple answers)		
Family	136	21.9
Japanese friend	21	3.4
Vietnamese friend	59	9.5
People at the same office	54	8.7
People at the same school	15	2.4
Medical professionals (including through online consultation)	27	4.3
Religious facilities (temples, churches, etc.)	4	0.6
Other	23	3.7

The mean PHQ-9 score was 7.9 ± 6.0 points. Mild, moderate, severe, and very severe depressive symptoms were observed in 212 (34.1%), 117 (18.8%), 52 (8.4%), and 34 (5.5%) participants, respectively. Further, 203 (32.7%) individuals had a PHQ-9 score ≥ 10 points. The mean GAD-7 score was 5.4 ± 5.3 points. Mild, moderate, and severe symptoms of anxiety were observed in 169 (27.2%), 69 (11.1%), and 47 (7.6%) participants, respectively. Moreover, 285 (45.9%) individuals had a GAD-7 score ≥ 5 points (Table 4).

**Table 4:** Symptoms of depression and anxiety (n = 621)

	n	%
Depressive symptoms (PHQ-9)		
Mean ± SD	7.9 ± 6.0	
No depression (0–4 points)	206	33.2
Mild (5–9 points)	212	34.1
Moderate (10–14 points)	117	18.8
Severe (15–19 points)	52	8.4
Very severe (20–27 points)	34	5.5
10–27 points	203	32.7
Anxiety symptoms (GAD-7)		
Mean ± SD	5.4 ± 5.3	
No anxiety (0–4 points)	336	54.1
Mild (5–9 points)	169	27.2
Moderate (10–14 points)	69	11.1
Severe (15–21 points)	47	7.6
5–21 points	285	45.9

In the unadjusted bivariate model, several factors were found to be associated with depressive symptoms. These factors include age (odds ratio [OR]: 0.92, 95% confidence interval [CI]: 0.89–0.96), being single (OR: 0.45, 95% CI: 0.28–0.72), having a visa of residence based on employment (OR: 3.93, 95% CI: 1.16–13.24), high education level (OR: 1.54, 95% CI: 1.05–2.26), pre-existing health conditions (OR: 2.37, 95% CI: 1.60–3.51), low socioeconomic status (OR: 2.80, 95% CI: 1.93–4.07), and not having a partner with whom to discuss one's health (OR: 1.63, 95% CI: 1.11–2.38). Factors associated with anxiety included age (OR: 0.92, 95% CI: 0.89–0.96), duration of residence in Japan (OR: 0.92, 95% CI: 0.86–0.97), being single (OR: 2.31, 95% CI: 1.52–3.50), having a visa of residence based on employment (OR: 5.09, 95% CI: 1.73–14.96), high education level (OR: 1.83, 95% CI: 1.28–2.62), pre-existing health conditions (OR: 2.51, 95% CI: 1.69–3.73), low socioeconomic status (OR: 3.18, 95% CI: 2.26–4.48), and not having a partner with whom to discuss one's health (OR: 2.16, 95% CI: 1.51–3.09). In multivariable logistic regression model, depressive symptoms were associated with age (OR: 0.94, 95% CI: 0.89–0.99), pre-existing health conditions (OR: 2.46, 95% CI: 1.61–3.76), and low socioeconomic status (OR: 2.47, 95% CI: 1.64–3.71). Factors related to anxiety included being single (OR: 1.72, 95% CI: 1.01–2.93), pre-existing health conditions (OR: 2.52, 95% CI: 1.63–3.88), low socioeconomic status (OR: 2.72, 95% CI: 1.87–3.97), and not having a partner with whom to discuss one’s health (OR: 1.66, 95% CI: 1.11–2.47) (Table 5).

**Table 5:** Factors associated with symptoms of depression and anxiety (n = 596)

	Depressive symptoms (PHQ-9)				Anxiety symptoms (GAD-7)			
	Unadjusted bivariate model		Multivariable logistic regression model		Unadjusted bivariate model		Multivariable logistic regression model	
	Odds ratio [95% CI]	p	Odds ratio [95% CI]	p	Odds ratio [95% CI]	p	Odds ratio [95% CI]	p
Age	0.92 [0.89–0.96]	< 0.001 **	0.94 [0.89–0.99]	0.043 *	0.92 [0.89–0.96]	< 0.001 **	0.97 [0.92–1.03]	0.348
Duration of residence in Japan	0.96 [0.90–1.02]	0.16	1.08 [0.99–1.17]	0.084	0.92 [0.86–0.97]	0.005 **	1.03 [0.95–1.12]	0.441
Sex								
Female	1.00 (reference)		1.00 (reference)		1.00 (reference)		1.00 (reference)	
Male	1.02 [0.73–1.43]	0.92	0.92 [0.63–1.34]	0.656	1.26 [0.92–1.73]	0.156	1.09 [0.76–1.57]	0.64
Marital status								
Married	1.00 (reference)		1.00 (reference)		1.00 (reference)		1.00 (reference)	
Single, divorced, or bereaved	0.45 [0.28–0.72]	0.001 **	1.61 [0.90–2.88]	0.109	2.31 [1.52–3.50]	< 0.001 **	1.72 [1.01–2.93]	0.044 *
Visa status								
Residence based on status and position	1.00 (reference)		1.00 (reference)		1.00 (reference)		1.00 (reference)	
Residence based on employment	3.93 [1.16–13.24]	0.03 *	2.46 [0.56–10.82]	0.233	5.09 [1.73–14.96]	0.003 **	2.51 [0.67–9.37]	0.172
Japanese language proficiency								
Ability to speak well/to some extent	1.00 (reference)		1.00 (reference)		1.00 (reference)		1.00 (reference)	
Barely able to speak	0.80 [0.44–1.44]	0.45	1.01 [0.50–2.00]	0.99	0.71 [0.41–1.23]	0.218	0.78 [0.40–1.53]	0.474
Education level								
College, University, or higher	1.00 (reference)		1.00 (reference)		1.00 (reference)		1.00 (reference)	
Technical school	1.35 [0.86–2.14]	0.192	1.16 [0.71–1.91]	0.549	1.23 [0.80–1.88]	0.354	1.05 [0.66–1.69]	0.831
High school or lower	1.54 [1.05–2.26]	0.027 *	0.94 [0.59–1.49]	0.784	1.83 [1.28–2.62]	0.001 **	1.20 [0.77–1.86]	0.423
Pre-existing health conditions								
No	1.00 (reference)		1.00 (reference)		1.00 (reference)		1.00 (reference)	
Yes	2.37 [1.60–3.51]	< 0.001 **	2.46 [1.61–3.76]	< 0.001 **	2.51 [1.69–3.73]	< 0.001 **	2.52 [1.63–3.88]	< 0.001 **
Subjective socioeconomic status								
General or better	1.00 (reference)		1.00 (reference)		1.00 (reference)		1.00 (reference)	
Difficult or very difficult	2.80 [1.93–4.07]	< 0.001 **	2.47 [1.64–3.71]	< 0.001 **	3.18 [2.26–4.48]	< 0.001 **	2.72 [1.87–3.97]	< 0.001 **
COVID-19 infection rate in the area of residence								
Low	1.00 (reference)		1.00 (reference)		1.00 (reference)		1.00 (reference)	0.654
Middle	0.84 [0.56–1.26]	0.399	0.97 [0.62–1.52]	0.895	0.72 [0.49–1.05]	0.088	0.83 [0.54–1.27]	0.391
High	1.08 [0.70–1.66]	0.724	1.11 [0.70–1.77]	0.663	0.94 [0.62–1.41]	0.762	0.96 [0.61–1.51]	0.859
Partner with whom to discuss one’s health								
Yes	1.00 (reference)		1.00 (reference)		1.00 (reference)		1.00 (reference)	
No	1.63 [1.11–2.38]	0.013 *	1.23 [0.81–1.88]	0.339	2.16 [1.51–3.09]	< 0.001 **	1.66 [1.11–2.47]	0.013 *

*CI* confidence interval

* p-value < 0.05

** p-value <0.01

## Discussion

This is the initial demonstration that about half of Vietnamese migrants in Japan exhibited symptoms of depression and anxiety amid the COVID-19 pandemic. Furthermore, the symptoms of depression and anxiety displayed significant associations with pre-existing health conditions and a low socioeconomic status in the multivariable regression analysis. Besides these associations, symptoms of depression were associated with younger age, and the symptoms of anxiety were associated with the absence of partners with whom to discuss one’s health. These results demonstrate that there is a mental health problem among Vietnamese migrants in Japan, highlighting the need for financial support and measures for preventing social isolation.

Our findings demonstrated that the presence of pre-existing health conditions significantly impacted mental health among Vietnamese migrants in Japan. A study conducted in South Korea during the COVID-19 pandemic revealed that migrants with a history of health issues experienced moderate to severe anxiety [14]. Similarly, a study conducted on Spanish migrants during the pandemic found that a history of previous mental illness was associated with depressive symptoms [15]. Another study conducted during the pandemic highlighted a history of depressive disorder as one of the major factors influencing mental health [16]. These findings suggest that pre-existing conditions, particularly pre-existing mental illness, may have influenced the mental health of migrants during the COVID-19 pandemic. Among the most important health issues in Japan is the health disparity among migrants living in Japan given the lack of access to medical services [17]. The COVID-19 pandemic further restricted access to medical services among migrants in Japan, including Vietnamese residents, which might contribute to increases in their anxiety.

Our analysis revealed that younger individuals have a slightly higher risk of experiencing depressive symptoms. In a study conducted in four countries in August and October 2020, age was found to be a protective factor for anxiety symptoms in Vietnam [18]. Similarly, a longitudinal study conducted in Japan between 2020 and 2021 reported that depressive symptoms were more severe in the young adult group (aged 20–39) compared to the middle-aged group, although the age groups differed from those in our study [19]. Previous research has indicated that older adults are better able to cope with stressors [20, 21], and this finding may be applicable to Vietnamese migrants in Japan as well.

Our analysis also revealed that being single and absence of partners with whom to discuss one’s health significantly affected the risk of experiencing symptoms of anxiety. The Immigration Services Agency of Japan reported that foreigners living in Japan had few connections with others outside of work [22]. A South Korean study reported that migrants who lived alone had moderate-to-extreme anxiety symptoms when compared with those living with their families [23]. These findings indicate that connections with others, especially family members, are important for maintaining mental health among migrants.

The present study had some limitations. The findings of this study should be interpreted with caution as they may not be applicable to the entire Vietnamese migrant population in Japan. The non-random recruitment process, reliance on Facebook for subject selection, and the high dropout rate among respondents are limitations that could affect the generalizability of the results. Additionally, the self-reported and retrospective nature of the data may have introduced recall bias, further impacting the reliability of the findings. Furthermore, the lack of prior study on the mental health status of Vietnamese migrants before COVID-19 pandemic prevented us from making comparisons with their mental health during the COVID-19 pandemic. Furthermore, it is important to note that participants who entered Japan after the pandemic may have responded to the question regarding "Monthly income when compared with that before COVID-19" by considering their wages earned in their home country. This factor should be considered when interpreting the results. However, this is the initial demonstration to observe a compromised mental health status among Vietnamese migrants in Japan during the COVID-19 pandemic.

## Conclusions

In this cross-sectional survey, we observed symptoms of depression and anxiety from the half of Vietnamese migrants in Japan. Secondly, we identified a significant association between symptoms of depression and anxiety and pre-existing health conditions and low socioeconomic status. Lastly, the absence of a partner for discussing health concerns was found to be associated with higher levels of anxiety symptoms. Increasing their social support and promptly implementing effective measures for their mental health is imperative.

## Data Availability

Not applicable.

## Abbreviations

CI: Confidence interval
GAD-7: Generalized anxiety disorder-7
OR: Odds ratio
PHQ-9: Patient Health Questionniare-9
SD: Standard deviation
